# Determination of amphetamines, ketamine and their metabolites in hair with high-speed grinding and solid-phase microextraction followed by LC-MS

**DOI:** 10.1080/20961790.2020.1838403

**Published:** 2020-12-07

**Authors:** Liang Meng, Yong Dai, Chen Chen, Jun Zhang

**Affiliations:** a Department of Forensic Science, Fujian Police College, Fuzhou, China; b Engineering Research Center, Fujian Police College, Fuzhou, China; c Department of Forensic Science, Sichuan Police College, Luzhou, China; d Drug Research Center of Integrated Traditional Chinese and Western Medicine, Affiliated Traditional Chinese Medicine Hospital of Southwest Medical University, Luzhou, China

**Keywords:** Forensic sciences, forensic toxicology, drugs of abuse, high-speed grinding, hair analysis, liquid chromatography-mass spectrometry, solid-phase microextraction

## Abstract

A novel hair sample pre-treatment method based on high-speed grinding and solid-phase microextraction (SPME) had been applied for the determination of amphetamines, ketamine and their metabolites in hair samples by liquid chromatography-mass spectrometry (LC-MS). A 20 mg sample of hair was ground with 2 mL of saturated sodium carbonate solution using a high-efficiency hair grinder with 70 Hz oscillation for 2 min at 4 °C. After centrifuging, 1.5 mL of the supernatant was transferred and treated with SPME by direct immersion (DI-SPME). The target analytes extracted by fibre were desorbed and analysed using LC-MS. Under the optimum conditions, a recovery of 90.2%–95.8% was obtained for all analytes. The analytical method was linear for all analytes in the range from 0.2 to 10 ng/mg with the correlation coefficient ranging from 0.9985 to 0.9993. The detection limits for all analytes were estimated to be 0.067 ng/mg. The accuracy (mean relative error) was within ±6.9% and the precision (relative standard error) was less than 6.8%. The combination of high-speed grinding of hair and SPME had the advantages of being easy to perform, environment-friendly and high in detection sensitivity. The proposed method offered an alternative analytical approach for the sensitive detection of drugs in hair samples for forensic purposes.

**Key Points**
- The SPME was involved for the determination of drugs in hair with LC-MS.
- The hair high-speed grinding combined with SPME was firstly developed.
- Good linearity, sensitivity, recovery and precision were achieved.

## Introduction

Synthetic drugs, such as amphetamines and ketamine, are the most widely abused drugs in China now. Due to the central excitatory and psychedelic effect, they have become popular among young people for recreational usage [1–5]. Drug users often take a mixture of drugs to experience the synergistic stimulation produced by various drugs and reduce anxiety and stress. As a consequence, the acute poisoning or pathological damage to multiple systems and organs of the body caused by overdose had happened occasionally [6–10]. In recent years, “Shenxianshui”, a kind of oral solution containing a variety of drugs [3] or instant solid “milk tea” were popular in some entertainment venues in China [4]. This new way of camouflage or abuse was very enticing, making more young people fall into the abyss of drug abuse; meanwhile, the camouflage form of ordinary beverages makes it more difficult for the law enforcement to crack down on drug crimes. In our previous work, these beverages were found to contain amphetamines, ketamine and other psychotropic drugs, as well as new psychoactive substances [3,4].

In China, hair has become a new biological matrix for the identification of drug addiction since the Ministry of Public Security issued *Amending the Measures for Identification of Drug Addiction* in 2017 [11], which clearly stipulated that hair samples should be used for testing, thus providing a scientific basis for law enforcement to identify drug abuse. Compared with blood, urine, saliva or other biological samples, hair samples have the advantages of stability, easy access, and long detection time window, and are considered to be ideal for identifying drug abuse [12–17]. Nevertheless, traditional hair pre-treatment methods (acid or alkali hydrolysis) are complicated, time-consuming and often associated with low analyte recovery and cross-contamination [13,18–20]. On the contrary, the high-speed grinding could completely break up the hair cuticles, which facilitates the release of drugs and their metabolites in the medulla of hair for liquid chromatography-mass spectrometry (LC-MS/MS) analysis [17,21–24]. However, due to the low concentration of drugs and their metabolites in hair and the presence of interferent (endogenous substances), false positive or false negative results are difficult to avoid [25,26]. In order to obtain accurate qualitative and quantitative results, it is necessary to develop analytical methods with higher sensitivity and accuracy.

In the present study, high-speed grinding, solvent extraction and solid-phase microextraction (SPME) were combined together to extract the target analytes in hair samples for LC-MS analysis. The aim of this study was to develop and validate the method for the determination of the popular drugs of abuse in China, i.e. ketamine, norketamine, methamphetamine (MA), amphetamine (AM), 3,4-methylenedioxymethamphetamine and 3,4-methylenedioxyamphetamine in hair samples.

## Materials and methods

### Reagents and materials

MA hydrochloride, AM sulphate, 3,4-methylenedioxymethamphetamine (MDMA) hydrochloride, 3,4-methylenedioxyamphetamine (MDA) hydrochloride, ketamine hydrochloride and norketamine hydrochloride reference standards were purchased from Institute of Forensic Science (99%, China Ministry of Public Security, Beijing). Methamphetamine-*d*_5_ (MA-*d*_5_, 1.0 mg/mL in methanol, certified reference material, isotopic purity: 0.00% D_0_ to D_2_, 0.01% D_3_, 2.55% D_4_, 97.44% D_5_), MDMA-*d*_5_ (1.0 mg/mL in methanol, certified reference material, isotopic purity: 0.00% D_0_ to D_2_, 0.04% D_3_, 4.33% D_4_, 95.63% D_5_) and ketamine-*d*_4_ (1.0 mg/mL in methanol, certified reference material, isotopic purity: 0.00% D_0_, 0.00% D_1_, 0.03% D_2_, 3.22% D_3_, 96.15% D_4_, 0.61% D_5_) were purchased from Cerilliant (Darmstadt, Germany) for internal standard (IS). Methanol, acetonitrile and formic acid, all of high performance liquid chromatography (HPLC) grade, were purchased from Fluka (St. Louis, MO, USA). Sodium carbonate and all other analytical grade reagents used for experiments were purchased from Sinopharm (Beijing, China). All standard solutions (1 mg/mL) were prepared with methanol and stored in the refrigerator at 4 °C prior to use.

Three different SPME fibres were acquired from Sigma-Aldrich-Supelco (Bellefonte, PA, USA), i.e. 85 µm film of polypropylene (PP), 100 µm film of polydimethylsiloxane (PDMS) and a 60 µm film of PDMS/divinylbenzene (DVB). These fibres were tested for their capacity to extract analytes. The analyses were performed using fibre holder where the fibres were protected.

Blank hair samples donated from healthy Chinese volunteers (students in our college: five males and five females) were cut at the posterior-vertex region as close to the scalp as possible and stored at room temperature in an envelope. The authentic hair samples were obtained from the Fujian Police College Judicial Expertise Center (Fuzhou, China), which were proved drug-positive with Standards of the Ministry of Justice of China [27].

### Apparatus

The analysis was performed on an Agilent-1200 series HPLC system coupled to an Agilent G6130A single quadrupole MS instrument (Agilent Technologies, Santa Clara, CA, USA). The separation of the extracted compounds was carried out on an Agilent Poroshell EC-C18 column (2.1 mm × 100.0 mm, 1.9 µm) at 40 °C. The mobile phase was methanol (solvent A) and 0.1% formic acid aqueous solution (solvent B), respectively. Gradient elution was performed at 0.3 mL/min with a total run time of 15.0 min: 20% B held for 1 min, 1–4 min, 20%*B*→5%B and held for 4 min; 8–8.1 min, 5%*B*→20%B; and finally held for 6.9 min.

The electrospray ionization (ESI) was set at positive ion mode. Source conditions were set as follows: drying gas (N_2_) flow 10 L/min; drying gas temperature 350 °C; capillary voltage 3.0 kV. The instrument was operated in the scan mode in the range from 50–750 (*m/z*) for qualitative analysis and selected ion monitoring (SIM) mode for quantitative analysis with the parameters described in Table 1.

**Table 1.** MS conditions for the determination of analytes and IS by LC-MS.

Analytes	Select ions (*m/z*)	Collision energy (eV)
Methamphetamine	150.1, 91.1	15
Amphetamine	136.1, 119.1	17
Methamphetamine-*d*_5_	155.2, 91.1	15
MDMA	194.2, 163.4	15
MDA	180.1, 163.1	11
MDMA-*d*_5_	199.1, 163.4	15
Ketamine	238.1, 179.1	18
Norketamine	224.1, 207.1	15
Ketamine-*d*_4_	242.1, 183.1	18

internal standard: IS; LC-MS: liquid chromatography-mass spectrometry; MDMA: 3,4-methylenedioxymethamphetamine; MDA: 3,4-methylenedioxyamphetamine.

High-efficiency hair grinder (JXFSTPRP-CL; Jingxin, Shanghai, China) was used for hair samples preparation.

### Hair sample pre-treatment and SPME process

The hair sample was washed once with 1 mL of acetone and twice with 0.5 mL of water and shaken for 1 min, dried overnight at room temperature prior to the analysis [28]. And then the hair sample was cut into segments 3 mm long. A 20 mg hair segments were weighed into a 5 mL centrifuge tube (containing 10 yttria-stabilized zirconia beads to improve hair grinding efficiency, 3.2 mm × 8, 5.2 mm × 2), and 2.0 mL saturated sodium carbonate solution (contains IS at 50 ng/mL) was added. After that, the centrifugal tube was placed into high-efficiency hair grinder for grinding. The hair cuticle was completely broken after high-frequency oscillation with 70 Hz for 2 min at 4 °C, and the target analytes were extracted into the solution.

The tube was then centrifuged for 3 min at 10 625× *g* and 4 °C. A 1.5 mL of the supernatant was transferred to a 2-mL sample bottle, in which a magnetic stirrer (5 mm in diameter) was placed. The bottle was sealed and placed on the SPME sampling bench for stirring and heating at 60 °C. Simultaneously, the SPME fibre was immersed into the sample solution for 15 min. After that, the fibre was desorbed in the desorption chamber for 1 min. Then the valve was switched from the load position to inject position and the analytes were transferred to the chromatographic column.

### Method validation

Method validation was based on published recommendations [29,30]. The linearity of the method was assessed in spiked samples. The six calibration curves at 10 ng, 20 ng, 50 ng, 100 ng, 200 ng, 500 ng target analytes were added to 20 mg blank hair and then treated in accordance with the above process (*n* = 5). Based on the experimental data, the correlation of analytes concentration (*x*) with the peak area ratio of analytes to IS (*y*) was obtained. Linearity of the calibration curve was calculated using a line of best fit with correlation factor expected to be >0.99. Limit of detection (LOD) was calculated as three times of the signal-to-noise ratio (S/N). Lower limit of quantification (LLOQ) was calculated as 10 times of the signal to noise ratio. The deviation of the measured concentration from the nominal concentration should be within ±15% except for the LLOQ level where a ±20% deviation is acceptable.

Intra- and inter-day (over 5 days) precision and accuracy studies were investigated using quality control samples which were blank samples spiked with analytes at 20, 50 and 200 ng (*n* = 5). Accuracy was deemed acceptable if the calculated concentrations fell within 20% of the concentration spiked (expressed as a percentage mean relative error (MRE)). Precision was deemed acceptable if the percentage relative standard deviation (RSD) was <15%. Recovery percentage of the proposed method referred to comparing the peak area of the proposed method treated analyte spiked sample with the pure analyte standard at the equivalent concentration without going through the proposed process.

## Results and discussion

### Optimization of hair grinding conditions

The drugs and their metabolites exist in the hair medulla tightly wrapped by the thicker outer cuticle, which needs to be pre-treated with high-speed physical grinding to completely break the cuticle, so that the drugs and their metabolites in the hair medulla could be extracted into the solution. There were several methods described in the literature utilizing an ultrasonic bath to extract the drugs out of the hair. Most procedures involved hair pulverization followed by ultrasonication for several hours to extract the drugs from the hair [17,20–23]. In the present study, hair pulverization and solvent extraction were combined together with high-speed grinding using a high-efficiency hair grinder, followed by a 15 min SPME desorption procedure. This procedure is more convenient and time-saving when compared with the previous method [17,20–23].

The factors including grinding time, oscillation frequency and grinding bead, were investigated. The extraction efficiencies of analytes increased with the increase of oscillation frequency and grinding time. However, a longer grinding time could cause a higher temperature, which might lead to the decomposition or volatilization of target analytes. As a compromise, the oscillation frequency was set at 70 Hz, and the grinding time was 2 min.

### Optimization of SPME conditions

#### Optimization of SPME fibre

**Figure 1.**
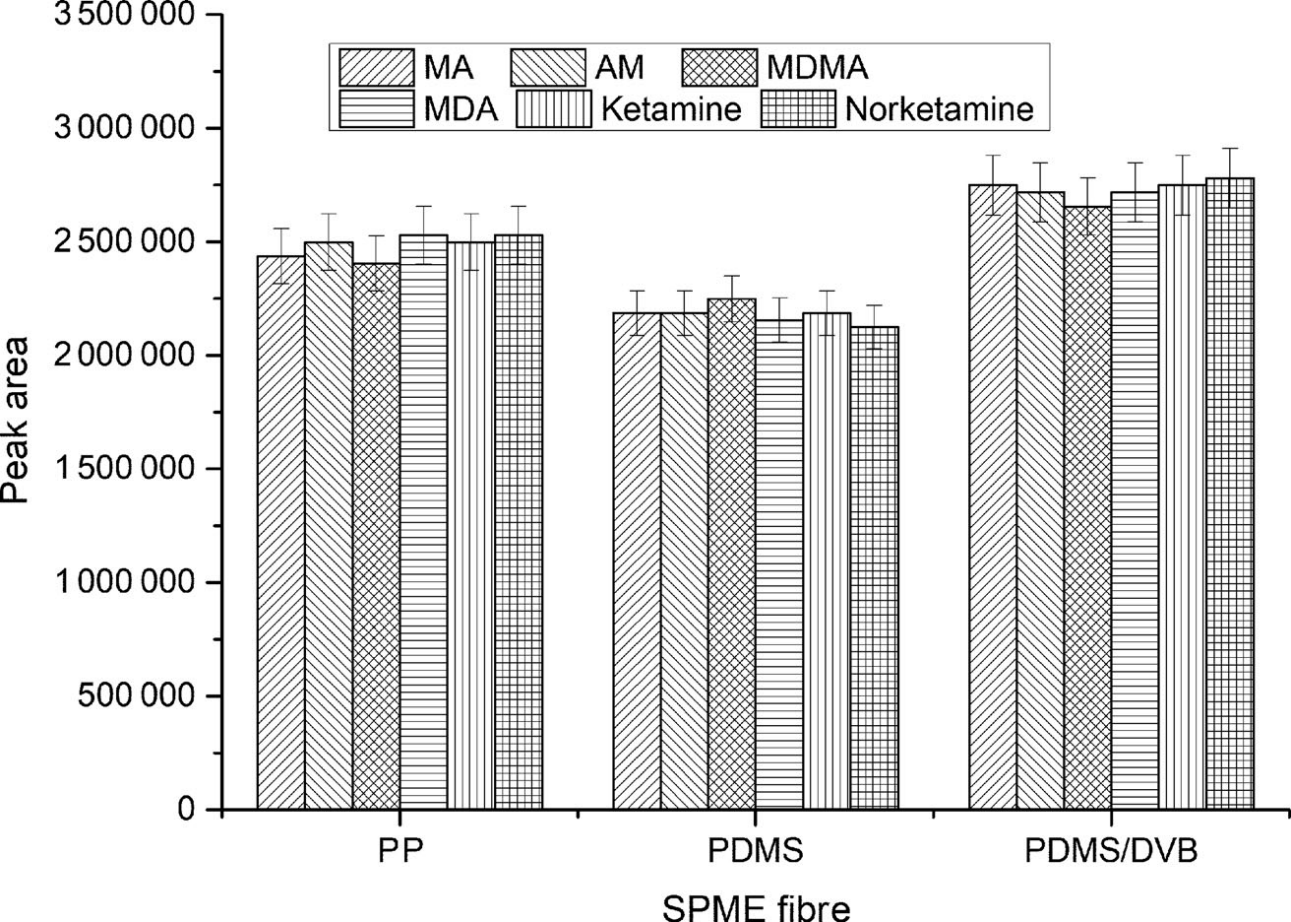
Effect of types of solid-phase microextraction (SPME) fibre on extraction efficiency conditions—extraction solvent: saturated sodium carbonate solution (pH 12), extraction temperature: 60 °C, extraction time: 15 min. Concentration of analytes: 4 ng/mg. MA: methamphetamine; AM: amphetamine; MDMA: 3,4-methylenedioxymethamphetamine; MDA: 3,4-methylenedioxyamphetamine; PP: polypropylene; PDMS: polydimethylsiloxane; DVB: divinylbenzene.

SPME has been used to extract drugs out of the hair [31–33] or body fluid [34–36] with GC-MS [31–33] or LC-MS [37,38]. In the present study, SPME combined with LC-MS was applied to extract and analyse three drugs and their metabolites from the hair samples. Three different fibres suitable for HPLC with different polarities were investigated, including 85 µm film of PP (suitable for strong polar analytes), 100 µm film of PDMS (suitable for weak polar analytes) and 60 µm film of PDMS/DVB (suitable for medium polar analytes). The results showed that PDMS/DVB fibre performed better in extraction efficiency, as seen in Figure 1. The probable reason was that the polarity of PDMS/DVB was between the PP and PDMS, suitable for the extraction of amine compounds.

#### Optimization of extraction temperature and time

**Figure 2.**
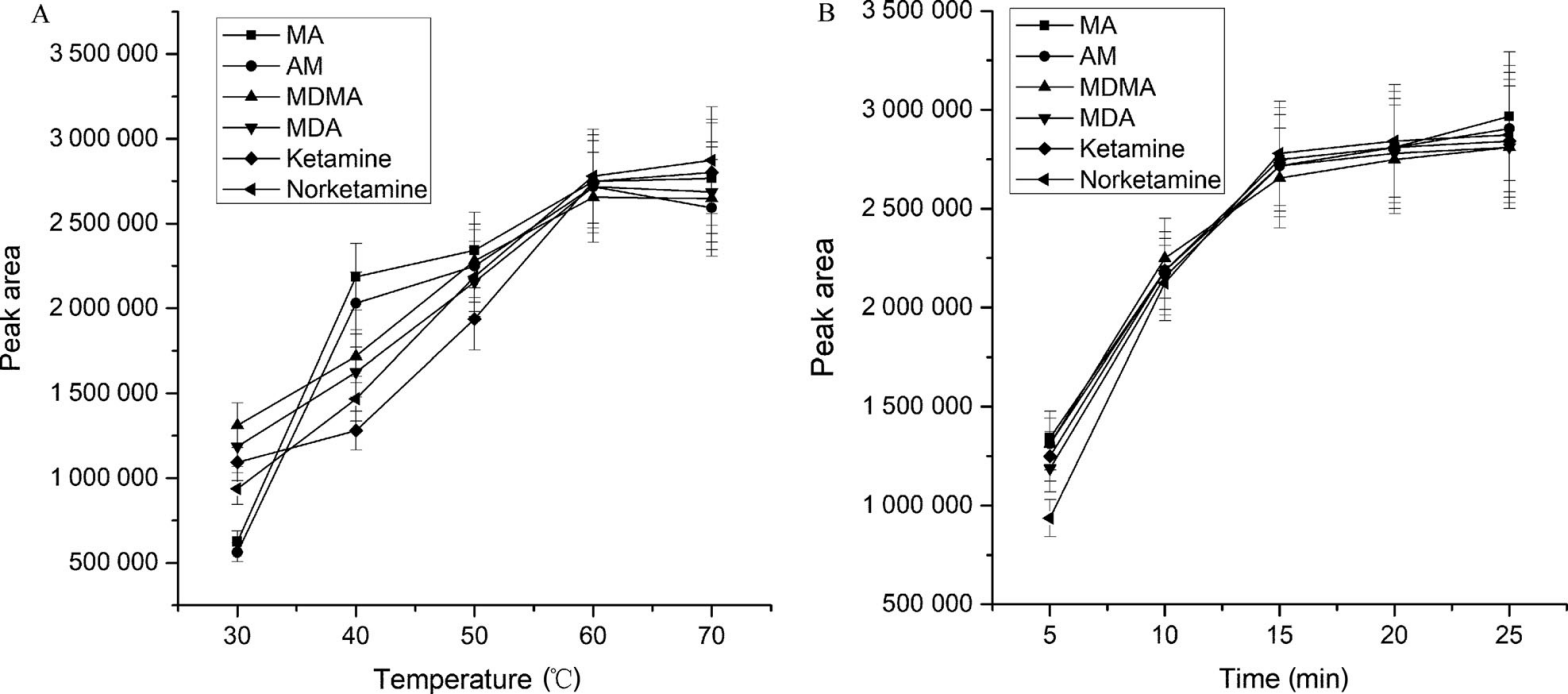
Effect of extraction temperature (A) and time (B) on extraction efficiency conditions. (A) solid-phase microextraction (SPME) fibre: polydimethylsiloxane/divinylbenzene (PDMS/DVB), extraction solvent: saturated sodium carbonate solution (pH 12), extraction time: 15 min; (B) SPME fibre: PDMS/DVB, extraction solvent: saturated sodium carbonate solution (pH 12), extraction temperature: 60 °C. Concentration of analytes: 4 ng/mg. MA: methamphetamine; AM: amphetamine; MDMA: 3,4-methylenedioxymethamphetamine; MDA: 3,4-methylenedioxyamphetamine.

Extraction temperature had a great influence on the extraction efficiency. The temperature could change the partition coefficient of the target analytes between solution and extraction medium, thus affecting the adsorption efficiency of extraction fibre. The thermal motion of target analytes in the solution increased with the increasing of temperature, which caused an increased molecular diffusion probability to the extraction fibre. Nonetheless, a higher temperature would disrupt the partition equilibrium, and the back diffusion might lead to a sharp reduction in extraction efficiency. The effect of temperature (at 30 °C, 40 °C, 50 °C, 60 °C and 70 °C) on extraction efficiency was investigated. The peak areas of analytes increased with the increase of temperature. As seen in Figure 2(A), the optimized extraction temperature for each analyte was different due to their different structures. Thus, the extraction temperature was chosen at 60 °C to suit the majority of analytes.

The distribution equilibrium time of analyte in the sample solution and extraction medium was affected by its physical and chemical properties (such as chemical structures, partition coefficient), sample matrix and adsorption properties of extraction medium. It would cause inadequate adsorption with a short extraction time, as the distribution equilibrium would not be reached, leading to a low extraction efficiency. The long extraction time would cause the loss of adsorptive analytes by the extraction medium. The effect of extraction time (at 5, 10, 15, 20 and 25 min) on extraction efficiency was investigated. The experimental results showed the peak areas of analytes increased with the increase in extraction time, and peaked at around 15 min, as seen in Figure 2(B). Thus, the extraction time was selected to be 15 min.

#### Effect of ionic strength and the pH of the sample solution

**Figure 3.**
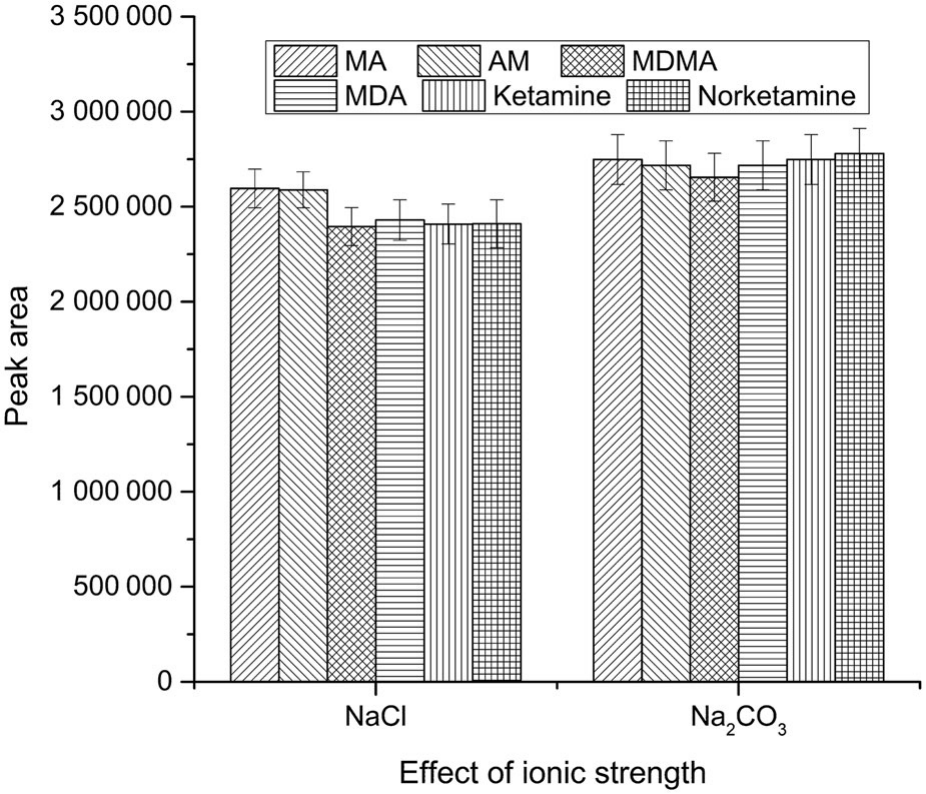
Effect of ionic strength on extraction efficiency conditions—solid-phase microextraction (SPME) fibre: polydimethylsiloxane/divinylbenzene (PDMS/DVB), extraction temperature: 60 °C, extraction time: 15 min, pH 12. Concentration of analytes: 4 ng/mg. MA: methamphetamine; AM: amphetamine; MDMA: 3,4-methylenedioxymethamphetamine; MDA: 3,4-methylenedioxyamphetamine.

The ionization degree and solubility of analytes were reduced with the increase in ionic strength of sample solution, thus improving the adsorption efficiency of the extraction fibre. NaCl and Na_2_CO_3_ were examined in this study. The results showed that the extraction efficiencies were improved with the addition of Na_2_CO_3_ to the extraction solution, as can be seen in Figure 3. Meanwhile, since the analytes were all alkaline substances, increasing the pH value of the extraction solution was conducive to the analytes to be uncharged and to be extracted effectively [39]. Saturated Na_2_CO_3_ solution could not only provide enough ionic strength, but also stabilize the pH value of extraction system at 12. Therefore, saturated Na_2_CO_3_ solution was used as the hair pre-treatment solution in this experiment.

### Evaluation of the proposed method

**Figure 4.**
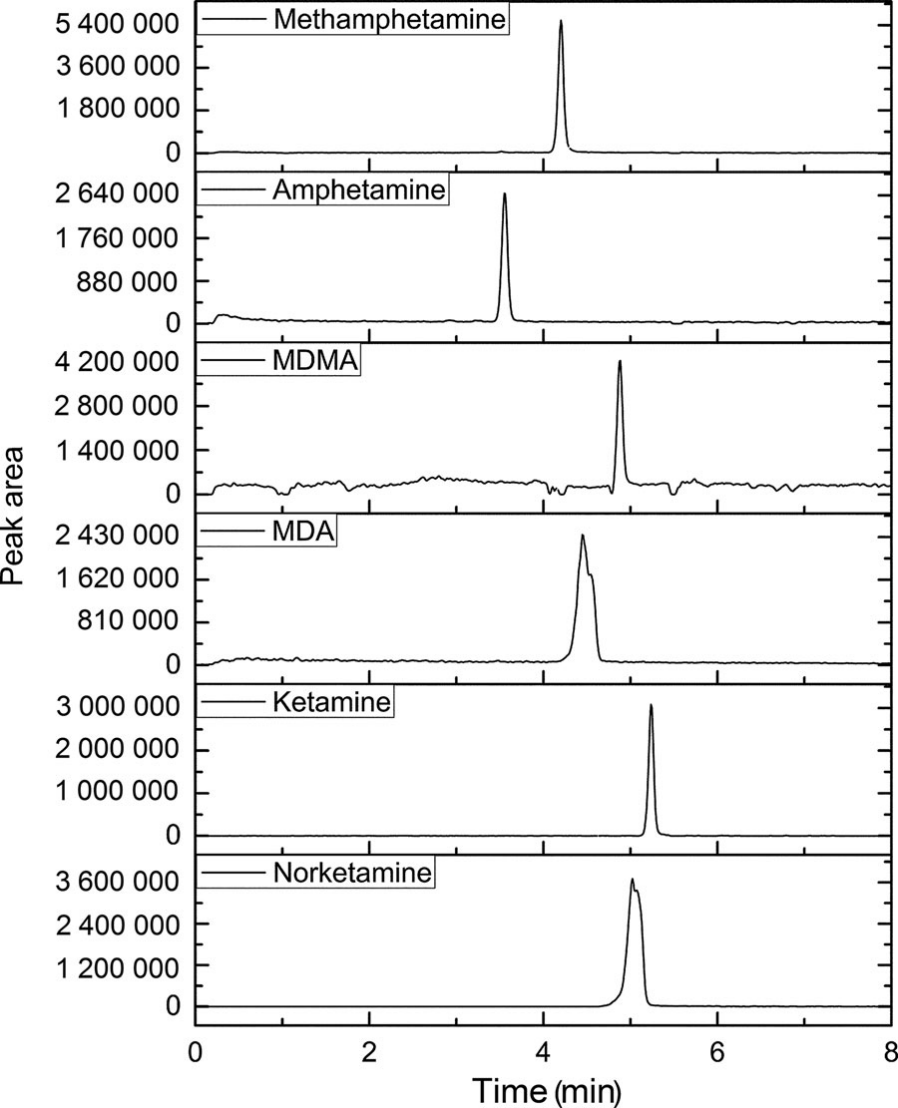
Chromatogram for target analytes spiked sample in SIM mode conditions—SPME fibre: PDMS/DVB, extraction solvent: saturated sodium carbonate solution (pH 12), extraction time: 15 min, extraction temperature: 60 °C. Concentration of analytes: 4 ng/mg. MDMA: 3,4-methylenedioxymethamphetamine; MDA: 3,4-methylenedioxyamphetamine.

Under the optimized conditions, the linear range, detection limit, precision, repeatability and recovery of the method were investigated using spiked hair samples, with the results shown in Table 2. Typical chromatogram obtained for the spiked sample under the optimum conditions is shown in Figure 4. The experimental results showed that the acceptable linear relationships in the range of 0.1–10.1 ng/mg were achieved with the correlation coefficient (*r*) ranging from 0.9985 to 0.9993. The LODs were estimated to be 0.067 ng/mg. The LLOQs were found to be 0.2 ng/mg. In the analysis of the spiked samples, recoveries of 90.1%–95.8% were obtained. Precision and accuracy at the three concentration levels were determined to be satisfactory (Table 2). The results indicate that the proposed method has high sensitivity and good reproducibility and holds the potential to be applied to the analysis of target analytes in biological samples.

**Table 2.** The performance of the proposed methods in spiked samples.

Analytes	Regression equation	Linear dynamic range (ng/mg)	*r*	LOD (ng/mg)	Added (ng/mg)	Recovery (%)	RSD (%), *n* = 5		MRE (%), *n* = 5	
							Intra-day	Inter-day	Intra-day	Inter-day
Methamphetamine	*y* = 0.0158*x* – 0.034	0.1–10.0	0.9990	0.067	0.5	95.4	5.7	5.1	3.3	6.2
					2.0	93.3	5.4	4.3	6.5	3.4
					10.0	94.7	3.0	2.2	5.8	2.4
Amphetamine	*y* = 0.0214*x* – 0.0235	0.1–10.0	0.9993	0.067	0.5	91.4	3.4	4.3	4.6	3.2
					2.0	94.3	3.3	3.8	4.3	1.9
					10.0	94.3	2.1	3.7	6.2	1.7
MDMA	*y* = 0.0196*x* – 0.0195	0.1–10.0	0.9989	0.067	0.5	89.9	6.2	4.3	6.3	3.8
					2.0	91.3	1.6	2.3	5.3	6.5
					10.0	90.9	3.7	4.2	3.4	5.3
MDA	*y* = 0.0123*x* – 0.0405	0.1–10.0	0.9985	0.067	0.5	92.7	6.2	5.9	4.6	4.4
					2.0	93.6	2.3	3.6	6.9	3.0
					10.0	94.4	4.7	6.4	5.2	2.9
Ketamine	*y* = 0.0177*x* – 0.0415	0.1–10.0	0.9992	0.067	0.5	94.1	6.8	5.6	4.5	5.8
					2.0	93.3	3.6	5.0	5.1	5.8
					10.0	95.2	6.3	6.6	1.9	6.0
Norketamine	*y* = 0.0249*x* – 0.0900	0.1–10.0	0.9991	0.067	0.5	94.4	3.9	5.9	3.6	5.0
					2.0	95.8	2.9	3.9	3.3	4.2
					10.0	90.1	5.5	6.1	3.5	2.1

LOD: limit of detection; RSD: relative standard deviation; MRE: mean relative error; MDMA: 3,4-methylenedioxymethamphetamine; MDA: 3,4-methylenedioxyamphetamine.

### Analysis of real hair samples

The proposed method described above was applied to determine the drugs in real human hair samples. The hair sample (0–3 cm) was cut at the posterior-vertex region as close to the scalp as possible from the suspected drug addicts during police operations, and analysed with the proposed method. There were five drug-positive samples obtained from four males and one female for the study. As can be seen in Table 3, the concentrations of target analytes in the hair samples were ranged from 1.9–7.9 ng/mg for MA, 0.8–3.2 ng/mg for AM, 3.7–9.7 ng/mg for ketamine and 3.6–8.6 ng/mg for norketamine, respectively. The four male subjects had a natural black hair colour. The female subject had dyed hair, while ketamine and norketamine were also detected. The results were in agreement with the ones obtained previously when the hair drug testing procedure described in the Standards of the Ministry of Justice of China was followed [27], which indicates that the proposed method can be applied to the real hair sample analysis for drugs of abuse testing.

**Table 3.** The analytic result of real sample with the proposed methods.

Samples	Property descriptions		Proposed method/standards^a^ (ng/mg)			
	Age (yr)	Colour	MA	AM	Ketamine	Norketamine
Male 1	30	Black	5.4/5.6	1.2/1.0	9.7/10.1	8.6/8.3
Male 2	27	Black	2.3/2.2	0.8/1.0	3.7/3.5	4.1/4.0
Male 3	41	Black	1.9/2.4	–	4.6/5.0	3.6/4.3
Male 4	38	Black	7.9/7.4	3.2/3.0	–	–
Female 1	36	Dyed brown	–	–	8.2/7.8	7.4/6.4

^a^The results were obtained from the Fujian Police College Judicial Expertise Centre (Fuzhou, China). MA: methamphetamine; AM: Amphetamine.

## Conclusion

In the present study, high-speed grinding and SPME pre-treatment coupled to LC-MS was used for the extraction and analysis of amphetamines, ketamine and their metabolites in hair samples. Hair grinding and solvent extraction were carried out simultaneously prior to further purification and extraction using SPME. The proposed method has the benefits of being simple, environment-friendly, quick to perform and high in detection accuracy and sensitivity. The method offers an alternative analytical tool for the sensitive detection of drugs in hair for judicial or medico-legal purposes.

## References

[1] Su S, Fairley CK, Mao L, et al. Estimates of the national trend of drugs use during 2000–2030 in China: a population-based mathematical model. Addict Behav. 2019;93:65–71. 30685570 10.1016/j.addbeh.2019.01.022

[2] Ji Kwon N, Han E. A review of drug abuse in recently reported cases of driving under the influence of drugs (DUID) in Asia, USA, and Europe. Forensic Sci Int. 2019;302:109854. 31255839 10.1016/j.forsciint.2019.06.012

[3] Zhu B, Meng L, Zheng K. Inspection and analysis of mixed drugs recently seized in China. Forensic Sci Int. 2014;242:e44–e47. 25106067 10.1016/j.forsciint.2014.07.013

[4] Zhu B, Meng L, Zheng K. Analysis of milk tea as a new mixed drug substance in China. J Forensic Sci Med. 2017;3:18–21.

[5] Bao Y-P, Liu Z-M, Li J-H, et al. Club drug use and associated high-risk sexual behaviour in six provinces in China. Addiction. 2015;110:11–19. 10.1111/add.12770 25533860

[6] McDougall SA, Rios JW, Apodaca MG, et al. Effects of dopamine and serotonin synthesis inhibitors on the ketamine-, D-amphetamine-, and cocaine-induced locomotor activity of preweanling and adolescent rats: sex differences. Behav Brain Res. 2020;379:112302. 31655095 10.1016/j.bbr.2019.112302 PMC6917827

[7] Pearson-Dennett V, Faulkner PL, Collie B, et al. Use of illicit amphetamines is associated with long-lasting changes in hand circuitry and control. Clin Neurophysiol. 2019;130:655–665. 30870801 10.1016/j.clinph.2019.02.005

[8] Wang Z, Han S, Cai M, et al. Environmental behavior of methamphetamine and ketamine in aquatic ecosystem: degradation, bioaccumulation, distribution, and associated shift in toxicity and bacterial community. Water Res. 2020;174:115585. 32105996 10.1016/j.watres.2020.115585

[9] Wai MSM, Chan WM, Zhang AQ, et al. Long-term ketamine and ketamine plus alcohol treatments produced damages in liver and kidney. Hum Exp Toxicol. 2012;31:877–886. 22354085 10.1177/0960327112436404

[10] Sun H-Q, Chen H-M, Yang F-D, et al. Epidemiological trends and the advances of treatments of amphetamine-type stimulants (ATS) in China. Am J Addict. 2014;23:313–317. 24724890 10.1111/j.1521-0391.2014.12116.x

[11] Decision on Amending the Measures for Identification of Drug Addiction. Beijing (China): the Ministry of Public Security of the People's Republic of China and the National Health and Family Planning Commission of the People's Republic of China. Decree No. 142. Available from: http://www.gov.cn/gongbao/content/2017/content_5213189.htm. Chinese.

[12] Zhuo Y, Wang X, Wu J, et al. Simultaneous quantitative determination of amphetamines, opiates, ketamine, cocaine and metabolites in human hair: application to forensic cases of drug abuse. J Forensic Sci. 2020;65:563–569. 31498435 10.1111/1556-4029.14179

[13] Wu Y-H, Lin K-l, Chen S-C, et al. Integration of GC/EI-MS and GC/NCI-MS for simultaneous quantitative determination of opiates, amphetamines, MDMA, ketamine, and metabolites in human hair. J Chromatogr B Analyt Technol Biomed Life Sci. 2008;870:192–202. 10.1016/j.jchromb.2008.06.017 18585989

[14] Madry MM, Kraemer T, Baumgartner MR. Large scale consumption monitoring of benzodiazepines and z-drugs by hair analysis. J Pharm Biomed Anal. 2020;183:113151. 32092690 10.1016/j.jpba.2020.113151

[15] Shin Y, Kong TY, Cheong JC, et al. Simultaneous determination of 75 abuse drugs including amphetamines, benzodiazepines, cocaine, opioids, piperazines, zolpidem and metabolites in human hair samples using liquid chromatography-tandem mass spectrometry. Biomed Chromatogr. 2019;33:e4600. 31116452 10.1002/bmc.4600

[16] Meier U, Colledge F, Imfeld S, et al. Distribution pattern of common drugs of abuse, ethyl glucuronide, and benzodiazepines in hair across the scalp. Drug Test Anal. 2019;11:1522–1541. 31407516 10.1002/dta.2679

[17] Xiang P, Shen M, Drummer OH. Review: drug concentrations in hair and their relevance in drug facilitated crimes. J Forensic Leg Med. 2015;36:126–135. 26454219 10.1016/j.jflm.2015.09.009

[18] Su P-H, Chang Y-Z, Yang C, et al. Perinatal effects of combined use of heroin, methadone, and amphetamine during pregnancy and quantitative measurement of metabolites in hair. Pediatr Neonatol. 2012;53:112–117. 22503258 10.1016/j.pedneo.2012.01.008

[19] Wu Y-H, Lin K-L, Chen S-C, et al. Simultaneous quantitative determination of amphetamines, ketamine, opiates and metabolites in human hair by gas chromatography/mass spectrometry. Rapid Commun Mass Spectrom. 2008;22:887–897. 18288687 10.1002/rcm.3409

[20] Shen M, Xiang P, Wu H, et al. Detection of antidepressant and antipsychotic drugs in human hair. Forensic Sci Int. 2002;126:153–161. 12084493 10.1016/s0379-0738(02)00051-8

[21] Wang R, Xiang P, Yu Z, et al. Application of hair analysis to document illegal 5-methoxy-N,N-dissopropyltrptamine (5-MeO-DiPT) use. Forensic Sci Int. 2019;304:109972. 31604205 10.1016/j.forsciint.2019.109972

[22] Wang T, Shen B, Wu H, et al. Disappearance of R/S-methamphetamine and R/S-amphetamine from human scalp hair after discontinuation of methamphetamine abuse. Forensic Sci Int. 2018;284:153–160. 29408724 10.1016/j.forsciint.2018.01.011

[23] Ji J-j, Yan H, Xiang P, et al. An LC-MS/MS method for the simultaneous determination of 12 psychotropic drugs and metabolites in hair: identification of acute quetiapine poisoning using hair root. Forensic Sci Int. 2019;301:341–349. 31202147 10.1016/j.forsciint.2019.05.040

[24] Tabernero MJ, Felli ML, Bermejo AM, et al. Determination of ketamine and amphetamines in hair by LC/MS/MS. Anal Bioanal Chem. 2009;395:2547–2557. 19806348 10.1007/s00216-009-3163-4

[25] Favretto D, Vogliardi S, Tucci M, et al. Occupational exposure to ketamine detected by hair analysis: a retrospective and prospective toxicological study. Forensic Sci Int. 2016;265:193–199. 27017567 10.1016/j.forsciint.2016.03.010

[26] Ou J, Zhang Y, Chen S, et al. An evaluation of the cut-off value of methamphetamine in hair samples *via* HPLC-MS/MS. Forensic Sci Int. 2020;306:110094. 31864115 10.1016/j.forsciint.2019.110094

[27] Detection of 15 illicit drugs and metabolites in hair by liquid chromatography-tandem mass spectrometry. Beijing (China): Ministry of Justice of the People's Republic of China. Standard No. SF/Z JD0107025—2018. Available from: http://www.ssfjd.com/Files/jsgf/6/21.pdf. Chinese.

[28] Nielsen MKK, Johansen SS. Internal quality control samples for hair testing. J Pharm Biomed Anal. 2020;188:113459. 32659675 10.1016/j.jpba.2020.113459

[29] Meng L, Zhu B, Zheng K, et al. Ultrasound-assisted low-density solvent dispersive liquid-liquid microextraction for the determination of 4 designer benzodiazepines in urine samples by gas chromatography-triple quadrupole mass spectrometry. J Chromatogr B Analyt Technol Biomed Life Sci. 2017;1053:9–15. 10.1016/j.jchromb.2017.04.008 28399467

[30] Peters FT, Drummer OH, Musshoff F. Validation of new methods. Forensic Sci Int. 2007;165:216–224. 16781833 10.1016/j.forsciint.2006.05.021

[31] Aleksa K, Walasek P, Fulga N, et al. Simultaneous detection of seventeen drugs of abuse and metabolites in hair using solid phase micro extraction (SPME) with GC/MS. Forensic Sci Int. 2012;218:31–36. 22047752 10.1016/j.forsciint.2011.10.002

[32] Lucas ACS, Bermejo AM, Tabernero MJ, et al. Use of solid-phase microextraction (SPME) for the determination of methadone and EDDP in human hair by GC–MS. Forensic Sci Int. 2000;107:225–232. 10689574 10.1016/s0379-0738(99)00165-6

[33] Sporkert F, Pragst F. Use of headspace solid-phase microextraction (HS-SPME) in hair analysis for organic compounds. Forensic Sci Int. 2000;107:129–148. 10689567 10.1016/s0379-0738(99)00158-9

[34] Godage NH, Cudjoe E, Neupane R, et al. Biocompatible SPME fibers for direct monitoring of nicotine and its metabolites at ultra trace concentration in rabbit plasma following the application of smoking cessation formulations. J Chromatogr A. 2020;1626:461333. 32797819 10.1016/j.chroma.2020.461333

[35] Olszowy P, Szultka M, Fuchs P, et al. New coated SPME fibers for extraction and fast HPLC determination of selected drugs in human blood. J Pharm Biomed Anal. 2010;53:1022–1027. 20674211 10.1016/j.jpba.2010.07.002

[36] Filipiak W, Bojko B. SPME in clinical, pharmaceutical, and biotechnological research — how far are we from daily practice? TrAC Trends Anal Chem. 2019;115:203–213.

[37] Goryński K, Kiedrowicz A, Bojko B. Development of SPME-LC-MS method for screening of eight beta-blockers and bronchodilators in plasma and urine samples. J Pharm Biomed Anal. 2016;127:147–155. 26971030 10.1016/j.jpba.2016.03.001

[38] Zheng M-M, Wang S-T, Hu W-K, et al. In-tube solid-phase microextraction based on hybrid silica monolith coupled to liquid chromatography-mass spectrometry for automated analysis of ten antidepressants in human urine and plasma. J Chromatogr A. 2010;1217:7493–7501. 20980013 10.1016/j.chroma.2010.10.002

[39] Meng L, Zhang W, Meng P, et al. Comparison of hollow fiber liquid-phase microextraction and ultrasound-assisted low-density solvent dispersive liquid-liquid microextraction for the determination of drugs of abuse in biological samples by gas chromatography-mass spectrometry. J Chromatogr B Analyt Technol Biomed Life Sci. 2015;989:46–53. 10.1016/j.jchromb.2015.02.039 25801996

